# Dimethyl 4,4′-(diazenedi­yl)dibenzoate at 100 K

**DOI:** 10.1107/S1600536813026846

**Published:** 2013-10-05

**Authors:** Katarzyna Gajda, Bartosz Zarychta, Andrzej A. Domański, Krzysztof Ejsmont

**Affiliations:** a Faculty of Chemistry, University of Opole, Oleska 48, 45-052 Opole, Poland

## Abstract

In the asymmetric part of the unit cell of the title compound, C_16_H_14_N_2_O_4_, there are two chemically equivalent but crystallographic independent half mol­ecules. The geometric centre of each complete mol­ecule lies on a crystallographic inversion centre. Both mol­ecules are almost planar [mean deviations of atoms in the two molecules are 0.032 (2) and 0.044 (2) Å] and their geometries are similar. In the crystal, mol­ecules are arranged in columns along the *a* axis. There are no inter­molecular donor–acceptor distances shorter than 3.4 Å.

## Related literature

For general background to the use of azo compounds as dyes, pigments and advanced materials, see: Allmann (1997 ▶); Scott *et al.*, (2002 ▶); Maniam *et al.* (2008 ▶); Zeitouny *et al.*, (2009 ▶). For a related structure, see: Yu & Liu (2009 ▶); Niu *et al.* (2011 ▶). For related literature, see: Onto *et al.* (1998 ▶). For the Cambridge Structural Database, see: Allen (2002 ▶).
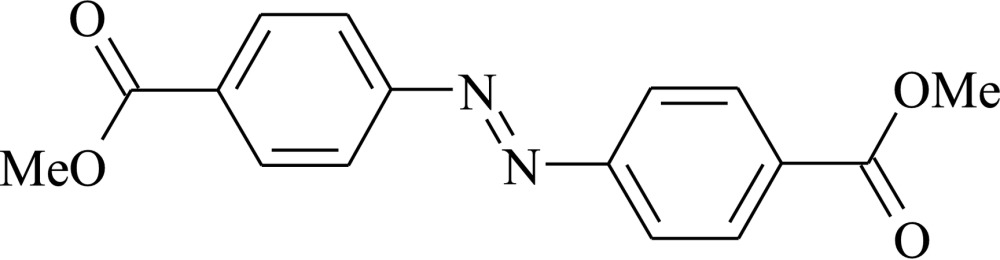


## Experimental

### 

#### Crystal data

C_16_H_14_N_2_O_4_
*M* *_r_* = 298.29
Triclinic, 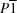

*a* = 3.8146 (8) Å
*b* = 11.2571 (18) Å
*c* = 16.904 (3) Å
α = 72.456 (16)°
β = 85.030 (18)°
γ = 84.468 (16)°
*V* = 687.6 (2) Å^3^
*Z* = 2
Mo *K*α radiation
μ = 0.11 mm^−1^
*T* = 100 K
0.35 × 0.17 × 0.15 mm

#### Data collection

Oxford Diffraction Xcalibur diffractometer
4264 measured reflections
2405 independent reflections
1652 reflections with *I* > 2σ(*I*)
*R*_int_ = 0.025

#### Refinement

*R*[*F* ^2^ > 2σ(*F* ^2^)] = 0.048
*wR*(*F* ^2^) = 0.147
*S* = 1.05
2405 reflections
213 parameters
H-atom parameters constrained
Δρ_max_ = 0.32 e Å^−3^
Δρ_min_ = −0.25 e Å^−3^

### 

Data collection: *CrysAlis CCD* (Oxford Diffraction, 2008 ▶); cell refinement: *CrysAlis RED* (Oxford Diffraction, 2008 ▶); data reduction: *CrysAlis RED*; program(s) used to solve structure: *SHELXS97* (Sheldrick, 2008 ▶); program(s) used to refine structure: *SHELXL97* (Sheldrick, 2008 ▶); molecular graphics: *SHELXTL* (Sheldrick, 2008 ▶); software used to prepare material for publication: *SHELXL97*.

## Supplementary Material

Crystal structure: contains datablock(s) global, I. DOI: 10.1107/S1600536813026846/fk2074sup1.cif

Structure factors: contains datablock(s) I. DOI: 10.1107/S1600536813026846/fk2074Isup2.hkl

Supplementary material file. DOI: 10.1107/S1600536813026846/fk2074Isup3.cml

Additional supplementary materials:  crystallographic information; 3D view; checkCIF report

## Figures and Tables

**Fig. 1.**
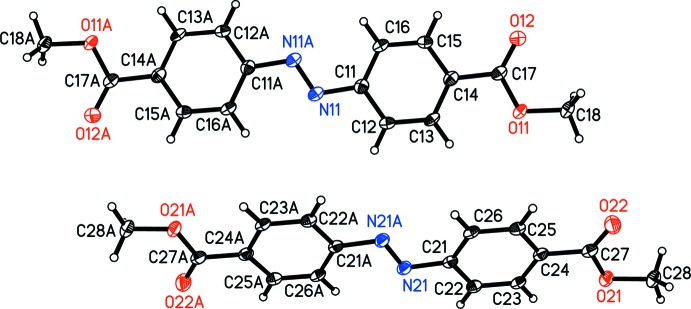
The molecular structure showing 50% displacement ellipsoids (arbitrary spheres for the H atoms). Symmetry code: A = -x, -y, -z.

**Fig. 2.**
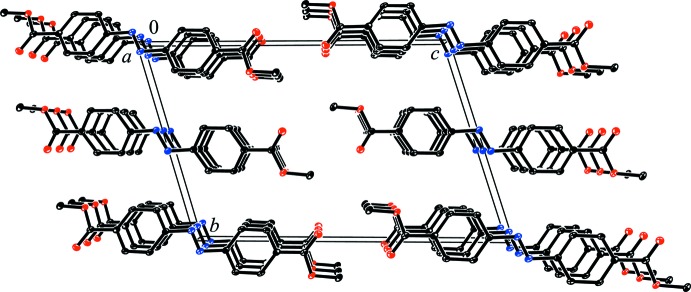
The packing diagram viewed along a-axis.

## supplementary crystallographic information

### 1. Comment

Azo compounds and its derivatives represent the dominant class of coloured compounds and are used as dyes and pigments (Allmann, 1997). Azobenzenes, due to its efficient and fully reversible photoisomerization and photoinduced anisotropy have widely been investigated as a component in photoresponsive materials (Zeitouny *et al.*, 2009).

There are two chemically equivalent but crystallographic independent molecules in the unit cell (Figure 1), both lie on crystallographic inversion centres. The molecules are almost planar and their geometries are similar; differences do not exceed 0.2 Å for bond lengths, 2° for valence angles and 3° for torsion angles.

All bond distances and angles lie in expected ranges and are in good agreement with the geometry of other *para*-substituted azobenzene derivatives (Yu & Liu, 2009; Niu *et al.*, 2011 and Allen, 2002).

The crystal packing is shown in Fig. 2. The molecules form columns along the *a* axis, the distance between stacked molecules is equal to 3.8146 (8) Å. There are no intermolecular C–H···O/N distances shorter than 3.4 Å.

### 2. Experimental

The compound was prepared according to literature procedure, (Onto *et al.*, 1998). Crystals of (I) suitable for X-ray crystal structure analysis was grown from benzene–n-heptane mixture (1:1).

### 3. Refinement

Apart from methyl hydrogens all H atoms were positioned geometrically, with C–H = 0.93 Å and *U*_iso_ (H) = 1.2*U*_eq_(C), methyl H atoms were derived from difference Fourier maps and refined as idealized groups with with C–H = 0.96 Å and *U*_iso_ (H) = 1.5*U*_eq_(C). All methyl-H atoms were allowed to rotate but not to tip.

### Crystal data

**Table:**

C_16_H_14_N_2_O_4_	*Z* = 2
*M**_r_* = 298.29	*F*(000) = 312
Triclinic, *P*1	*D*_x_ = 1.441 Mg m^−^^3^
Hall symbol: -P 1	Mo *K*α radiation, λ = 0.71073 Å
*a* = 3.8146 (8) Å	Cell parameters from 4264 reflections
*b* = 11.2571 (18) Å	θ = 3.6–25.0°
*c* = 16.904 (3) Å	µ = 0.11 mm^−^^1^
α = 72.456 (16)°	*T* = 100 K
β = 85.030 (18)°	Prism, red
γ = 84.468 (16)°	0.35 × 0.17 × 0.15 mm
*V* = 687.6 (2) Å^3^	

### Data collection

**Table:**

Oxford Diffraction Xcalibur diffractometer	1652 reflections with *I* > 2σ(*I*)
Radiation source: fine-focus sealed tube	*R*_int_ = 0.025
Graphite monochromator	θ_max_ = 25.0°, θ_min_ = 3.6°
Detector resolution: 1024 x 1024 with blocks 2 x 2 pixels mm^-1^	*h* = −4→2
ω–scan	*k* = −13→13
4264 measured reflections	*l* = −20→20
2405 independent reflections	

### Refinement

**Table:**

Refinement on *F*^2^	Primary atom site location: structure-invariant direct methods
Least-squares matrix: full	Secondary atom site location: difference Fourier map
*R*[*F*^2^ > 2σ(*F*^2^)] = 0.048	Hydrogen site location: inferred from neighbouring sites
*wR*(*F*^2^) = 0.147	H-atom parameters constrained
*S* = 1.05	*w* = 1/[σ^2^(*F*_o_^2^) + (0.0791*P*)^2^ + 0.205*P*] where *P* = (*F*_o_^2^ + 2*F*_c_^2^)/3
2405 reflections	(Δ/σ)_max_ < 0.001
213 parameters	Δρ_max_ = 0.32 e Å^−^^3^
0 restraints	Δρ_min_ = −0.25 e Å^−^^3^

### Special details

**Table:**

Geometry. All e.s.d.'s (except the e.s.d. in the dihedral angle between two l.s. planes) are estimated using the full covariance matrix. The cell e.s.d.'s are taken into account individually in the estimation of e.s.d.'s in distances, angles and torsion angles; correlations between e.s.d.'s in cell parameters are only used when they are defined by crystal symmetry. An approximate (isotropic) treatment of cell e.s.d.'s is used for estimating e.s.d.'s involving l.s. planes.
Refinement. Refinement of *F*^2^ against ALL reflections. The weighted *R*-factor *wR* and goodness of fit *S* are based on *F*^2^, conventional *R*-factors *R* are based on *F*, with *F* set to zero for negative *F*^2^. The threshold expression of *F*^2^ > σ(*F*^2^) is used only for calculating *R*-factors(gt) *etc*. and is not relevant to the choice of reflections for refinement. *R*-factors based on *F*^2^ are statistically about twice as large as those based on *F*, and *R*- factors based on ALL data will be even larger.

### Fractional atomic coordinates and isotropic or equivalent isotropic displacement parameters (Å^2^)

**Table:**

	*x*	*y*	*z*	*U*_iso_*/*U*_eq_	
O11	0.3301 (4)	0.15744 (14)	0.33935 (9)	0.0251 (4)	
O12	0.1772 (4)	−0.04021 (15)	0.39802 (10)	0.0296 (4)	
N11	0.0393 (5)	0.05005 (17)	0.00495 (12)	0.0216 (5)	
C11	0.0777 (6)	0.0436 (2)	0.08962 (14)	0.0191 (5)	
C12	0.2050 (6)	0.1480 (2)	0.10194 (14)	0.0196 (5)	
H12	0.2585	0.2160	0.0565	0.020 (6)*	
C13	0.2522 (5)	0.1511 (2)	0.18173 (14)	0.0191 (5)	
H13	0.3387	0.2208	0.1898	0.025 (6)*	
C14	0.1695 (5)	0.0493 (2)	0.25002 (14)	0.0194 (5)	
C15	0.0372 (6)	−0.0548 (2)	0.23750 (14)	0.0204 (5)	
H15	−0.0194	−0.1222	0.2830	0.020 (6)*	
C16	−0.0101 (6)	−0.0585 (2)	0.15834 (14)	0.0200 (5)	
H16	−0.0992	−0.1278	0.1504	0.028 (7)*	
C17	0.2225 (6)	0.0484 (2)	0.33681 (14)	0.0209 (5)	
C18	0.3974 (7)	0.1652 (2)	0.42107 (14)	0.0272 (6)	
H18A	0.4725	0.2464	0.4158	0.037 (7)*	
H18B	0.5788	0.1025	0.4448	0.031 (7)*	
H18C	0.1852	0.1519	0.4565	0.038 (8)*	
O21	0.6465 (4)	0.65469 (14)	0.35059 (10)	0.0248 (4)	
O22	0.8894 (4)	0.45639 (15)	0.38736 (10)	0.0308 (5)	
N21	0.4726 (5)	0.55083 (17)	0.00854 (12)	0.0206 (4)	
C21	0.5457 (6)	0.5432 (2)	0.09183 (14)	0.0184 (5)	
C22	0.4325 (6)	0.6478 (2)	0.11755 (14)	0.0193 (5)	
H22	0.3167	0.7168	0.0815	0.034 (7)*	
C23	0.4932 (5)	0.6485 (2)	0.19724 (13)	0.0188 (5)	
H23	0.4175	0.7183	0.2145	0.027 (7)*	
C24	0.6678 (6)	0.5450 (2)	0.25183 (14)	0.0194 (5)	
C25	0.7813 (5)	0.4400 (2)	0.22548 (14)	0.0196 (5)	
H25	0.8971	0.3710	0.2616	0.023 (6)*	
C26	0.7222 (6)	0.4383 (2)	0.14616 (14)	0.0193 (5)	
H26	0.7983	0.3686	0.1288	0.027 (7)*	
C27	0.7478 (6)	0.5448 (2)	0.33667 (14)	0.0212 (5)	
C28	0.7220 (7)	0.6663 (2)	0.43076 (14)	0.0279 (6)	
H28A	0.6386	0.7481	0.4340	0.046 (8)*	
H28B	0.9720	0.6542	0.4368	0.031 (7)*	
H28C	0.6053	0.6044	0.4744	0.039 (8)*	

### Atomic displacement parameters (Å^2^)

**Table:**

	*U*^11^	*U*^22^	*U*^33^	*U*^12^	*U*^13^	*U*^23^
O11	0.0366 (10)	0.0189 (9)	0.0242 (9)	−0.0057 (7)	−0.0049 (7)	−0.0110 (7)
O12	0.0411 (11)	0.0227 (10)	0.0250 (9)	−0.0081 (8)	−0.0030 (8)	−0.0052 (7)
N11	0.0227 (10)	0.0171 (10)	0.0270 (11)	−0.0007 (8)	−0.0018 (8)	−0.0096 (8)
C11	0.0183 (11)	0.0165 (12)	0.0233 (12)	0.0047 (9)	−0.0021 (9)	−0.0089 (9)
C12	0.0205 (11)	0.0136 (12)	0.0239 (13)	−0.0017 (9)	−0.0001 (9)	−0.0047 (9)
C13	0.0184 (11)	0.0133 (11)	0.0289 (13)	−0.0003 (9)	−0.0020 (9)	−0.0114 (10)
C14	0.0163 (11)	0.0185 (12)	0.0253 (13)	0.0014 (9)	−0.0019 (9)	−0.0100 (10)
C15	0.0232 (12)	0.0140 (11)	0.0228 (12)	−0.0005 (9)	−0.0017 (10)	−0.0039 (9)
C16	0.0198 (11)	0.0150 (12)	0.0274 (13)	−0.0027 (9)	−0.0005 (10)	−0.0094 (10)
C17	0.0203 (12)	0.0174 (12)	0.0272 (13)	−0.0014 (9)	−0.0013 (9)	−0.0101 (10)
C18	0.0355 (14)	0.0250 (14)	0.0268 (14)	−0.0061 (11)	−0.0062 (11)	−0.0141 (11)
O21	0.0352 (10)	0.0200 (9)	0.0231 (9)	−0.0018 (7)	−0.0041 (7)	−0.0115 (7)
O22	0.0428 (11)	0.0240 (10)	0.0263 (10)	0.0044 (8)	−0.0080 (8)	−0.0092 (8)
N21	0.0230 (10)	0.0177 (10)	0.0229 (10)	−0.0029 (8)	−0.0008 (8)	−0.0085 (8)
C21	0.0180 (11)	0.0156 (12)	0.0226 (12)	−0.0046 (9)	0.0015 (9)	−0.0069 (9)
C22	0.0188 (11)	0.0143 (11)	0.0245 (13)	−0.0006 (9)	−0.0020 (9)	−0.0055 (9)
C23	0.0189 (11)	0.0141 (11)	0.0255 (13)	−0.0034 (9)	0.0011 (9)	−0.0091 (9)
C24	0.0189 (11)	0.0177 (12)	0.0232 (12)	−0.0057 (9)	0.0017 (9)	−0.0079 (9)
C25	0.0182 (11)	0.0145 (12)	0.0258 (13)	−0.0012 (9)	−0.0017 (9)	−0.0055 (9)
C26	0.0201 (11)	0.0132 (11)	0.0270 (13)	−0.0021 (9)	−0.0003 (9)	−0.0093 (9)
C27	0.0217 (12)	0.0173 (12)	0.0263 (13)	−0.0045 (9)	0.0017 (10)	−0.0088 (10)
C28	0.0368 (14)	0.0263 (14)	0.0264 (14)	−0.0043 (11)	−0.0040 (11)	−0.0153 (11)

### Geometric parameters (Å, º)

**Table:**

O11—C17	1.345 (3)	O21—C27	1.342 (3)
O11—C18	1.456 (3)	O21—C28	1.456 (3)
O12—C17	1.215 (3)	O22—C27	1.216 (3)
N11—N11^i^	1.255 (3)	N21—N21^ii^	1.257 (4)
N11—C11	1.431 (3)	N21—C21	1.434 (3)
C11—C12	1.391 (3)	C21—C22	1.393 (3)
C11—C16	1.409 (3)	C21—C26	1.411 (3)
C12—C13	1.387 (3)	C22—C23	1.389 (3)
C12—H12	0.9300	C22—H22	0.9300
C13—C14	1.398 (3)	C23—C24	1.401 (3)
C13—H13	0.9300	C23—H23	0.9300
C14—C15	1.398 (3)	C24—C25	1.403 (3)
C14—C17	1.495 (3)	C24—C27	1.491 (3)
C15—C16	1.379 (3)	C25—C26	1.385 (3)
C15—H15	0.9300	C25—H25	0.9300
C16—H16	0.9300	C26—H26	0.9300
C18—H18A	0.9600	C28—H28A	0.9600
C18—H18B	0.9600	C28—H28B	0.9600
C18—H18C	0.9600	C28—H28C	0.9600
			
C17—O11—C18	116.32 (17)	C27—O21—C28	116.58 (18)
N11^i^—N11—C11	114.3 (2)	N21^ii^—N21—C21	114.3 (2)
C12—C11—C16	120.0 (2)	C22—C21—C26	120.4 (2)
C12—C11—N11	115.69 (19)	C22—C21—N21	115.86 (19)
C16—C11—N11	124.25 (19)	C26—C21—N21	123.76 (19)
C13—C12—C11	120.2 (2)	C23—C22—C21	119.7 (2)
C13—C12—H12	119.9	C23—C22—H22	120.2
C11—C12—H12	119.9	C21—C22—H22	120.2
C12—C13—C14	119.88 (19)	C22—C23—C24	120.5 (2)
C12—C13—H13	120.1	C22—C23—H23	119.8
C14—C13—H13	120.1	C24—C23—H23	119.8
C15—C14—C13	119.8 (2)	C23—C24—C25	119.5 (2)
C15—C14—C17	118.83 (19)	C23—C24—C27	121.7 (2)
C13—C14—C17	121.41 (19)	C25—C24—C27	118.8 (2)
C16—C15—C14	120.7 (2)	C26—C25—C24	120.5 (2)
C16—C15—H15	119.7	C26—C25—H25	119.8
C14—C15—H15	119.7	C24—C25—H25	119.8
C15—C16—C11	119.42 (19)	C25—C26—C21	119.4 (2)
C15—C16—H16	120.3	C25—C26—H26	120.3
C11—C16—H16	120.3	C21—C26—H26	120.3
O12—C17—O11	123.6 (2)	O22—C27—O21	123.7 (2)
O12—C17—C14	124.4 (2)	O22—C27—C24	124.4 (2)
O11—C17—C14	111.96 (18)	O21—C27—C24	111.90 (19)
O11—C18—H18A	109.5	O21—C28—H28A	109.5
O11—C18—H18B	109.5	O21—C28—H28B	109.5
H18A—C18—H18B	109.5	H28A—C28—H28B	109.5
O11—C18—H18C	109.5	O21—C28—H28C	109.5
H18A—C18—H18C	109.5	H28A—C28—H28C	109.5
H18B—C18—H18C	109.5	H28B—C28—H28C	109.5
			
N11^i^—N11—C11—C12	172.7 (2)	N21^ii^—N21—C21—C22	−169.5 (2)
N11^i^—N11—C11—C16	−8.4 (3)	N21^ii^—N21—C21—C26	10.9 (3)
C16—C11—C12—C13	1.2 (3)	C26—C21—C22—C23	−0.2 (3)
N11—C11—C12—C13	−179.92 (18)	N21—C21—C22—C23	−179.82 (18)
C11—C12—C13—C14	−0.4 (3)	C21—C22—C23—C24	0.1 (3)
C12—C13—C14—C15	−0.4 (3)	C22—C23—C24—C25	0.0 (3)
C12—C13—C14—C17	178.97 (19)	C22—C23—C24—C27	177.82 (19)
C13—C14—C15—C16	0.5 (3)	C23—C24—C25—C26	0.1 (3)
C17—C14—C15—C16	−178.93 (19)	C27—C24—C25—C26	−177.85 (19)
C14—C15—C16—C11	0.3 (3)	C24—C25—C26—C21	−0.1 (3)
C12—C11—C16—C15	−1.1 (3)	C22—C21—C26—C25	0.2 (3)
N11—C11—C16—C15	−179.91 (19)	N21—C21—C26—C25	179.82 (19)
C18—O11—C17—O12	0.8 (3)	C28—O21—C27—O22	1.5 (3)
C18—O11—C17—C14	−178.63 (18)	C28—O21—C27—C24	−178.16 (17)
C15—C14—C17—O12	4.4 (3)	C23—C24—C27—O22	177.8 (2)
C13—C14—C17—O12	−175.0 (2)	C25—C24—C27—O22	−4.3 (3)
C15—C14—C17—O11	−176.20 (18)	C23—C24—C27—O21	−2.5 (3)
C13—C14—C17—O11	4.4 (3)	C25—C24—C27—O21	175.33 (19)

Symmetry codes: (i) −*x*, −*y*, −*z*; (ii) −*x*+1, −*y*+1, −*z*.
